# The university students’ self-regulated effort, flexibility and satisfaction in distance education

**DOI:** 10.1186/s41239-022-00342-w

**Published:** 2022-07-22

**Authors:** Zeynep Turan, Sevda Kucuk, Sinem Cilligol Karabey

**Affiliations:** 1 Department of Computer Education & Instructional Technology, Kazim Karabekir Education Faculty, Ataturk University, 25240 Erzurum, Turkey; 2 Open and Distance Education Faculty, Ataturk University, 25240 Erzurum, Turkey

**Keywords:** Satisfaction, Self-regulation, Flexibility, University students, Distance education

## Abstract

Distance education offers flexible learning opportunities in terms of time, place and learning speed to teachers and students through internet technologies. However, the learning opportunities provided in distance education environments require students to act more autonomously and take more responsibility for regulating their learning processes to achieve their learning goals. For this reason, it is crucial to reveal the relationships between flexibility, self-regulated effort and satisfaction factors to provide efficient and effective learning environments in distance education. Accordingly, within the scope of this study, it is aimed to investigate university students’ perceptions of flexibility, self-regulated effort and satisfaction with the distance education process, and their views on distance education. Survey research was used as the research method in the study. The study sample consisted of 1760 university students studying at 28 different universities. Data were analysed using t-test, ANOVA, and regression methods. As a result of the study, male students were more satisfied with distance education than female students, and education faculty students had the highest level of satisfaction. In addition, self-regulated effort and flexibility variables were significant factors affecting students’ satisfaction towards distance education. Furthermore, the advantages and problems of distance education and suggestions for future distance learning environments were presented in the light of students views. Finally, the study’s implications and various recommendations for practitioners and researchers were discussed in detail.

## Introduction

Distance education is a method that allows students and teachers to come together and receive education through internet technologies, regardless of time and place, and is widely used today. This is especially true for higher education institutions, where the number of online courses and students registering for them has jumped (Wei & Chou, 2020). Additionally, the COVID-19 pandemic has necessitated significant changes in education processes worldwide, which has affected the education process of 1.6 billion students from 200 countries (UNESCO, 2020). Therefore, the entire world has urgently turned to distance education at all education levels to minimise the pandemic’s negative impacts on human health (Karadag et al., 2021). In this sense, universities have made an urgent transformation in their education processes with a sudden decision and gave all face-to-face classes through distance education. Distance education has been employed for a long time; however, it has become more widespread than ever before upon the COVID-19 pandemic.

One of the critical indicators of distance learning environments where effective learning processes take place is student satisfaction (Moore, 2005). It has been determined that student satisfaction in distance education is highly associated with students’ dropout rates, motivation, determination to complete an online course and success rates (Ali & Ahmad, 2011; Lo, 2010; Parlak, 2004; Yükseltürk & Yıldırım, 2008). In the literature, various research results show that distance education, when well designed, can provide as much or a higher level of student satisfaction as face-to-face education. In this context, in the meta-analysis study conducted by He et. al. (2021), studies comparing distance education and face-to-face education in the field of health sciences were examined. As a result of the study, It has been found that the students’ satisfaction studying in the distance education environments is higher than the students studying in face-to-face classroom environments. Also, according to Yen et. al. (2018), in their study comparing face-to-face, online and blended learning environments, students had equal satisfaction in all three learning environments.

Contrary to these findings, in the study conducted by Tratnik et. al. (2019), student satisfaction levels in face-to-face and online courses were compared in the English language teaching field. As a result, it was determined that students were more satisfied with face-to-face classes. In summary, it can be said that a well-designed distance learning environment is a course that emphasises interaction and active participation to facilitate learning, preferably while maintaining student flexibility (Driscoll et al., 2012). In addition, studies in the literature indicate that ensuring student satisfaction in distance education increases the success of both the institution and the student (Ali & Ahmad, 2011; Karataş & Üstündağ, 2008; Şahin, 2009). For this reason, revealing the factors affecting student satisfaction is very important for designing successful distance learning environments.

However, with the COVID-19 pandemic, the sudden transformation of face-to-face courses into distance education without any planning process has brought many negative situations in terms of student satisfaction. Indeed, Hodges et. al. (2020) stated that distance education carried out in any crisis is different from a typical distance education process. In this direction, in the literature, the distance education process, which is carried out in times of crisis without extensive preparation, as in the period of the COVID-19 pandemic, is expressed as “emergency remote teaching” (Bozkurt & Sharma, 2020; Hodges et al., 2020). In the literature, numerous studies have been conducted on the emergency remote teaching process carried out during the COVID-19 pandemic. These studies have revealed that students experience various difficulties in time management, motivation, and independent learning while taking courses through the distance education method that they are not used to before, and the quality of education they take is impaired (Lee et al., 2021; Means & Neisler, 2021; Weidlich & Kalz, 2021). Furthermore, the studies examining students’ satisfaction with this process have also indicated that students are not very satisfied with emergency remote teaching (Karadag et al., 2021; Simsek et al., 2021; Turan & Gürol, 2020). When considering such widespread use of distance education globally and the low satisfaction of students, the requirement of scientific research on distance education processes to design effective learning environments is undeniable.

## Literature review

Various factors affect the success of distance education. One of these factors is students’ satisfaction with distance education. In this sense, the studies in the literature have reported that satisfaction is one of the critical factors determining the success of online courses and is intertwined with many other factors (Hamdan et al., 2021; Wei & Chou, 2020). In the literature, some studies have been conducted to investigate the factors affecting satisfaction in online education. For example, Chow and Shi (2014) found that university students’ perceptions of e-learning, including perceived flexibility and motivation, had significant effects on e-learning satisfaction. Sahin and Shelley (2008) stated that students’ having the skills to use online tools and considering online learning useful and flexible affected their satisfaction positively. In addition, a study conducted with 1232 university students in Vietnam revealed that the quality of e-learning services had a positive effect on student satisfaction and loyalty (Pham et al., 2019). Student satisfaction is one of the significant indicators for the success and quality of online programs, but it is not the only factor (Yukselturk & Yildirim, 2008). Therefore, it can be asserted that it is crucial to identify the influencing factors in the satisfaction of students in distance education.

Distance education requires students to be more independent and self-regulated. Pintrich (2000) defines self-regulated learning as the process by which learners actively and constructively set goals and regulate their cognition, motivation, and behaviour to achieve their learning goals. Therefore, it is of great importance for the success of the learning process that the students in distance education environments have the skills they have created on their own in controlling, managing and planning their learning activities compared to the students in face-to-face learning environments (Ally, 2004). One way to ensure that such strategies are sustained is to use a different technique, self-regulated effort, under the umbrella of self-regulated learning. Self-regulated effort refers to how determined and dedicated students are in coping with teaching tasks perceived as tough in the learning environment (Duncan & McKeachie, 2005). Moreover, distance education boosts students’ self-regulated learning strategies due to its autonomous nature. The self-regulated effort shown with these strategies contributes to their learning goals (Barnard et al., 2009; Zimmerman, 2008). For example, Puzziferro (2008) found a relationship between self-regulated effort and learning in his study with university students. Students who reported higher levels of self-regulated effort to manage academic tasks were more successful than those who reported lower levels of self-regulated effort. In the light of all these findings, it is possible to say that determining the factors related to students’ self-regulation effort is vital for a successful distance learning process.

Distance education offers students flexible learning opportunities in terms of time, place, and learning speed; however, it requires students to act more autonomously and take more responsibility for regulating learning processes to achieve their learning goals (Shearer & Park, 2018). It can be asserted that flexible learning is the core subject of distance education (Bates, 2001). Van den Brande (1993) defines flexible learning as enabling students to study whenever and how they want to. In this sense, it is predicted that the examination of flexibility, which has an important place in distance education, would contribute to distance education planning in the future. Flexibility in distance education covers all activities from attending classes to the end of the learning process for students and is a critical element in individualising the learning process (Bergamin et al., 2012). With its flexibility feature, distance education offers students the opportunity to receive individual or group education from any person and institution they want, whenever and wherever they want (Shearer & Park, 2018). Also, flexible learning environments facilitate behavioural engagement and interaction in the learning environment (Kariippanon et al., 2019). Additionally, studies in the literature indicate that distance education environments that offer students a high level of flexibility in terms of technology, pedagogy, learning resources and activities can increase the academic success of students with these features (Austerschmidt & Bebermeier, 2019; Bergamin et al., 2010; Soffer et al., 2019). Although there are many sub-types of the concept of flexibility in different sources, this study is based on the classification of Bergamin et. al. (2012). In this classification, there are sub-types of flexibility such as “Flexibility of time management”, “Flexibility of teacher contact”, and “Flexibility of content” (Bergamin et al., 2012). “Flexibility of time management” is about students deciding when to learn and learn at their own pace. “Flexibility of teacher contact” indicates students’ ease and communication possibilities to communicate with the course instructor. Another sub-type, “Flexibility of content”, refers to the opportunities for students to learn the content they want, whenever and wherever they want.

Studies in the literature have reported that distance learning environments contribute to students’ autonomy experiences (Barnard et al., 2009; Firat, 2016). In this context, it can be asserted that autonomy is essential in self-learning, and self-learning is vital for students to be successful in distance education and to take their learning responsibilities (Firat, 2016). Furthermore, students need to have self-regulated learning skills to participate effectively in learning activities in a flexible learning environment (Bergamin et al., 2012). Also, Schraw (2007) states that autonomous and flexible learning encourages self-regulation in students. In addition, studies in the literature have reported that when students find online courses flexible in distance education, this has significant effects on their levels of satisfaction with the course (Chow & Shi, 2014; Sahin & Shelley, 2008). Kokoç (2019), on the other hand, reported in his study that perceived flexibility of time and perceived flexibility related to content had significant positive effects on students’ behavioural engagement and academic performance. Furthermore, flexibility specifically addresses the needs of employed and adult students since it considers the diversity of their previous knowledge and life experiences and supports self-learning (Ammenwerth et al., 2019). Therefore, flexibility in distance education is vital for students to improve their online learning processes.

Distance education at universities, especially during the COVID-19 pandemic, was conducted with students with heterogeneous demographic characteristics in terms of gender and department. Although the literature includes several studies examining the effect of gender on distance education processes, inconsistent findings have been determined. For example, Alghamdi et. al. (2020) determined that although women had stronger self-regulation skills in online learning than men, men could use more learning strategies and better technical skills than women. Tosuntaş et. al. (2015) revealed that female students tended to be more willing to employ the system than male students in online learning systems, thus resulting in better learning performance. In their study, Zhang et. al. (2021) found that male graduates showed a better performance than female graduates in the distance education program. On the other hand, various related studies have reported that gender did not affect the distance learning processes of students (Nistor, 2013; Yu, 2021). When considering all these findings, it is predicted that examining the impacts of gender and department factors on the distance education process will guide the design of online education environments.

As distance education becomes more and more widespread, this raises various questions about how to design effective distance education environments for students. Thus, it is crucial to reveal the relationships between flexibility, self-regulated effort, and satisfaction to create efficient and effective learning environments in distance education. In this context, the distance education process carried out during the COVID-19 pandemic has been examined with the following research questions. In this sense, the research questions are as follows:

1. What is the level of university students’ perceptions of flexibility, self-regulated effort, and satisfaction in distance education?
  - Do university students’ perceptions of flexibility, self-regulated effort, and satisfaction in distance education differ significantly based on gender?
  - Do university students’ perceptions of flexibility, self-regulated effort, and satisfaction in distance education differ significantly based on the discipline/department?
2. Is there a significant correlation between university students’ perceptions of flexibility, self-regulated effort, and satisfaction in distance education?
3. Do the university students’ perceptions of flexibility and self-regulated effort predict their satisfaction in distance education?
4. How are university students’ experiences (suggestions, problems, advantages) regarding distance education?

## Method

The study was designed as survey research. Survey research is based on taking the opinions of a large group of people about a particular topic or issue (Fraenkel et al., 2012). This method is generally a scientific research method carried out to figure out the unique properties of a population (Johnson & Christensen, 2000). This study investigated the perceived flexibility, self-regulated effort, and satisfaction of university students in Turkey in distance education during the COVID-19 pandemic. Furthermore, their views on the distance education process were detected.

### Participants

The researchers employed an online survey to collect data from university students from different universities in Turkey and the Turkish Republic of Northern Cyprus (TRNC). With the onset of the Covid-19 epidemic, first-term emergency remote teaching applications were carried out in universities in Turkey, and unplanned distance education was generally offered. In the next semester, when the data of this study were collected, a more planned distance education period was provided to the students since it was determined in advance that the courses would be given by distance education. In this context, instructors were given seminars on distance education, and the technical infrastructure of universities was strengthened, enabling instructors to prepare for distance lessons at least three months ago. The courses were carried out through asynchronous materials presented through a specific learning management system (Moodle, Blackboard et al.) and synchronous course sessions held weekly by the instructors. Ethics committee approval of the study was obtained from the university where the researchers were affiliated. The students were subjected to the survey at the end of the fall semester of the 2020–2021 academic year. One thousand eight hundred twenty-five undergraduate students responded to the questionnaire presented within the scope of the research, and the data of 1760 undergraduate students were accepted as valid and complete. One thousand seven hundred sixty undergraduate students (n = 1212, 68.9% female; n = 548, 31.1% male) studying in 17 faculties of 28 universities, including 27 universities in different regions of Turkey and one university in the TRNC participated in the study. The students had an average of 20.97 years (SD = 3.23). In the study, the faculties were examined under four categories: Education, Social Sciences, Medical and Health Sciences, and Engineering and Natural Sciences. The students participating in the study were mostly from education faculties (n = 691, 39.3%), and these students were pre-service teachers in different departments. The students in the discipline of Medical and Health Sciences (n = 475, 27.0%) were receiving education at Medical, Dentistry, and Nursing faculties and Vocational School of Health Services.

On the other hand, the students in the Social Sciences discipline (n = 391, 22.2%) received education in Literacy, History, Religion, Law, Tourism, and Business departments. Additionally, the students in Engineering and Natural Sciences (n = 391, 22.2%) were studying at engineering faculties and natural sciences departments such as biology, chemistry, and physics. A great majority of the participants were first-year students (n = 925, 52.6%), followed by sophomore, senior, and junior students, respectively. Table 1 shows the demographic information of the participants.

**Table 1:** Demographic information of the participants (N = 1760)

Measure	*N*	%
Gender		
Female	1212	68.9
Male	548	31.1
Class level		
Freshman	925	52.6
Sophomore	345	19.6
Junior	216	12.3
Senior	274	15.6
Discipline		
Education	691	39.3
Medical and health sciences	475	27.0
Social sciences	391	22.2
Engineering and natural sciences	203	11.3

### Data collection tools

An online survey prepared by the researchers was used as a data collection tool in the study. Before completing the survey, the participants were asked to read and approve a consent form to participate in the study voluntarily. The first part of the survey included questions about the demographic characteristics of the participants. Its second part had 18 items in the 5-point Likert type (1: I strongly disagree…5: I strongly agree) to determine the participants’ perceived flexibility, self-regulated effort, and satisfaction. It is rated by taking the arithmetic average of the items in each factor, and the average arithmetic ranges are as follows; 1.00–1.80; “Strongly disagree”, 1.81–2.60; “Disagree”, 2.61–3.40; “Neutral”, 3.41–4.20; “Agree”, and 4.21–5.00; “Strongly agree”. Bergamin et. al. (2012) developed the perceived flexibility scale and adapted it into Turkish by Kokoç (2020). The scale consists of three factors; flexibility of time management (3 items), the flexibility of teacher contact (2 items), and flexibility of content (4 items).

The related factors accounted for 56.456% of the total variance. It was determined that the composite reliability coefficient for the overall scale was 0.91 and the Cronbach’s alpha internal consistency coefficient was 0.83. Lange and Costley’s (2018) study used the self-regulated effort scale. Although the original scale was a 10-point Likert, this study used a 5-point Likert version of the scale to comply with the other factors of the survey. The Cronbach’s alpha for the self-regulated effort was 0.73. The satisfaction scale was developed by Kuo et. al. (2013), and Cronbach’s alpha value for the scale’s reliability was α = 0.93. In the study, the Cronbach’s alpha values of the survey consisting of the factors in these scales were calculated as 0.92. Table 2 shows each factor in the survey, the items in these factors and the reliability scores obtained. The last part of the survey included an open-ended question to reveal the students’ views on the distance education process. Students’ experiences, suggestions, and other factors they want to say about the distance course process were questioned with an open-ended question.

**Table 2:** Factors, items and reliability values

Factors and reliability values	Items
*Flexibility of time management* α = 0.80	I can decide when I want to learn
	I can define my own learning pace
	I can repeat the subject matter at will
*Flexibility of teacher contact* α = 0.84	I can contact the teacher at any time
	There are different ways of contacting the teacher
*Flexibility of content* α = 0.82	I have a say regarding the focus of the topics class
	I can prioritise topics in my learning
	I can choose between different learning forms, including on-campus study, online study, and self-study
	I can study topics of special interest
*Self-regulated effort* α = 0.71	I often lose focus when I study, so I quit before I finish what I planned to do (reversed)
	I work to do well at school even if I get confused
	When coursework is unclear, I give up or only study the easy parts (reversed)
	Even when study materials are complex, I manage to keep working until I finish
*Satisfaction (SAT)* α = 0.94	Overall, I am satisfied with online education
	The courses contributed to my educational development
	I am satisfied with the level of interaction that happened in the courses
	I am satisfied with taking fully online courses in this process

### Data analysis

Quantitative data obtained from the study were analysed with descriptive and predictive methods in line with the research questions. First, the skewness and kurtosis values were utilised to determine whether they were normally distributed. Tabachnick and Fidell (2007) state that if the sample size is large, the normality assumption is not violated in the skewness and kurtosis values of ± 1.96. In this study, the values were between these critical values. Thus the normality assumption was met. An independent sample t-test was used to determine whether or not gender had a significant effect on factors. The test for normality, examining standardised skewness and kurtosis values, indicated the data were statistically normal. However, Levene’s F test revealed that the homogeneity of variance assumption was not met (p = 0.00). As such, the Welch’s F test was used to identify the differences based on four different disciplines. An alpha level of 0.05 was used for all subsequent analyses. Also, Tamhane T2 was used as the Post-Hoc test to detect the difference between the groups since the assumption about the homogeneity of variances could not be achieved. The correlation between the factors was determined using Pearson’s correlation method. Finally, linear regression was applied to reveal to what extent flexibility and self-regulated effort predicted satisfaction. The data met all assumptions (linearity, normality, multicollinearity, and independence) (Field, 2009). The qualitative data obtained from the survey’s open-ended question were analysed using the content analysis method, creating categories and codes.

The qualitative data obtained at the beginning of the analysis process was organised by one of the researchers in line with the purpose of the research and divided into conceptually meaningful codes. Then, by creating a code list, the relationship of each data with the relevant code was checked. After this stage, all codings’ common and similar features were gathered under themes. Finally, the themes created were reconsidered with the work of three researchers, discussed extensively, and the themes on which a common view was agreed were determined.

## Findings

### Descriptive statistics of key measures

Table 3 shows the mean, standard deviation, skewness, and kurtosis values for each factor examined within the scope of the study. The highest mean score was observed in the flexibility of time management (M = 3.88, SD = 1.05), which was followed by the flexibility of content (M = 3.80, SD = 0.97), the flexibility of teacher contact (M = 3.51, SD = 1.23), and self-regulated effort (M = 3.38, SD = 2.26), respectively. The lowest mean score was determined in satisfaction (M = 2.77, SD = 1.35).

**Table 3:** Means and standard deviations of key measures (*N* = 1760)

Measure	*M*	SD	Skewness	Kurtosis
Flexibility of time management (FTM)	3.88	1.05	− 0.852	0.060
Flexibility of teacher contact (FTC)	3.51	1.23	− 0.483	− 0.792
Flexibility of content (FC)	3.80	0.97	− 0.822	0.252
Self-regulated effort (SRE)	3.38	2.16	1.01	− 0.321
Satisfaction (SAT)	2.77	1.35	0.215	− 1.24

^*^p < 0.05

### Gender differences on key measures

Table 4 shows the results obtained from the independent sample t-test, which was carried out to determine whether or not gender affected university students’ flexibility, self-regulated effort, and satisfaction. Accordingly, it was found that gender had a significant effect on FTC (t(1758) = − 0.275, p < 0.05) and SAT (t(1758) = − 3.82, p < 0.01). However, it was determined that the effect size value of the results was small (Cohen, 1988).

**Table 4:** Gender differences on flexibility, self-regulated effort, and satisfaction (N = 1760)

	Gender	n	Mean	SD	df	t	p	d
FTM	Female	1212	3.88	1.038	1758	− 0.275	0.783	
	Male	548	3.89	1.082				
FTC	Female	1212	3.45	1.231	1758	− 2.96	0.003*	0.154
	Male	548	3.64	1.235				
FC	Female	1212	3.77	0.973	1758	− 1.35	0.176	
	Male	548	3.84	0.966				
SRE	Female	1212	3.36	1.008	1758	− 1.23	0.219	
	Male	548	3.43	1.015				
SAT	Female	1212	2.69	1.326	1758	− 3.82	0.000*	0.191
	Male	548	2.95	1.388				

^*^p < 0.05

### Discipline differences on key measures

Table 5 shows the students’ flexibility, self-regulated effort, and satisfaction levels according to the discipline they were studying in. The students of education discipline at FTM, FTC, FC, and SRE had the highest mean score. In terms of satisfaction, students in natural and engineering sciences obtained the highest mean score. According to the Welch's ANOVA results, there were significant differences among the disciplines. Therefore, the Post-Hoc test was applied to determine the disciplines causing the difference. Since Levene’s statistic was less than 0.05, the homogeneity of the variances could not be met. For this reason, the Tamhane T2 Post-Hoc test, used in case of heterogeneity of variances, was used.

**Table 5:** Means, standard deviations, and Welch’s ANOVA results (N = 1760)

Measure	Social (n = 391)		Natural & Eng. (n = 203)		Health (n = 475)		Education (n = 691)		F (3, 1756)	est. ω^2^
	M	SD	M	SD	M	SD	M	SD		
FTM	3.79	1.06	3.84	1.05	3.83	1.22	3.98	0.909	3.617*	0.004
FTC	3.55	1.22	3.39	1.30	3.34	1.34	3.63	1.13	5.814*	0.008
FC	3.74	0.949	3.68	0.968	3.64	1.16	3.97	0.806	12.808*	0.020
SRE	3.31	0.953	3.38	0.998	3.23	1.17	3.53	0.909	9.439*	0.014
SAT	2.55	1.36	2.87	1.36	2.80	1.44	2.84	1.27	4.570*	0.006

^*^p < 0.05

A Welch's ANOVA revealed a statistically significant difference in FTM between at least two groups, Welch’s F (3, 1756) = 3.617, p < 0.05, est. ω^2^ = 0.004. Tamhane T2 post hoc test for multiple comparisons found that the mean value of FTM was significantly different between Social and Education (p < 0.05). There was a statistically significant difference in FTC, Welch’s F (3, 1756) = 5.814, p < 0.05, est. ω^2^ = 0.008. FTC was significantly different between Health and Education (p < 0.05). There was a statistically significant difference in FC, Welch’s F (3, 1756) = 12.808, p < 0.05, est. ω^2^ = 0.020. FC was significantly different between Education and the other three disciplines (p < 0.05). There was a statistically significant difference in SRE, Welch’s F (3, 1756) = 9.439, p < 0.05, est. ω^2^ = 0.014. SRE was significantly different between Social and Education, and Health and Education (p < 0.05). Lastly, there was a statistically significant difference in SAT, Welch’s F (3, 1756) = 4.570, p < 0.05, est. ω^2^ = 0.006. SAT was significantly different between Social and Education, and Social and Natural & Engineering. However, the estimated omega squared values showed that the total variances in the dependent variables were quite low. The post hoc comparisons are presented in Table 6.

**Table 6:** Post-hoc comparisons (N = 1760)

Dependent variable	Comparison		Mean difference	SE	p_tamhane_
	Condition	Condition			
FTM	Social	Education	− 0.192*	0.064	0.016
FTC	Health	Education	− 0.287*	0.075	0.001
FC	Social	Education	− 0.228*	0.057	0.000
	Natural & Eng.	Education	− 0.290*	0.075	0.001
	Health	Education	− 0.324*	0.061	0.000
SRE	Social	Education	− 0.225*	0.059	0.001
	Health	Education	− 0.301*	0.064	0.000
SAT	Social	Education	− 0.292*	0.084	0.003
	Social	Natural & Eng.	− 0.319*	0.118	0.041

^*^p < 0.05

### Correlations among key measures

Table 7 shows the correlations between students’ flexibility, self-regulated effort, and satisfaction levels. The correlations ranged from + 0.405 to + 0.705 (all coefficients were significant, p < 0.01). The correlations between factors were moderate.

**Table 7:** Correlation matrix for key measures (*N* = 1760)

Measure	1	2	3	4	5
1. FTM	–				
2. FTC	0.461**	–			
3. FC	0.705**	0.536**	–		
4. SRE	0.491**	0.405**	0.566**	–	
5. SAT	0.529**	0.406**	0.530**	0.517**	–

**p < 0.01 (two-tailed)

### Regression model for satisfaction

Table 8 shows the data obtained from the regression analysis, which was carried out to determine to what extent self-regulated effort and flexibility predicted students’ satisfaction with distance education. The resulting model was significant and predicted satisfaction by 39% [R2 = 0.39, F (4, 1755) = 279.83, p < 0.001]. The self-regulated effort had the most important effect on satisfaction, which FTM followed. FC and FTC had less effect on the model.

**Table 8:** Predicting satisfaction from self-regulated effort and flexibility measures (N = 1760)

Criterion	Predictors	*b*	CI_95%_ for *b*		β	*r*	*sr*^2^
			Lower	Upper			
Satisfaction	SRE	0.362	0.302	0.423	0.271	0.270	0.048
	FTM	0.311	0.243	0.378	0.242	0.211	0.028
	FC	0.208	0.128	0.288	0.150	0.121	0.009
	FTC	0.113	0.065	0.162	0.104	0.109	0.007

Fit for model *R*^2^ = 0.39, *F* (4, 1755) = 279.83, *p* < 0.001

### Problems, advantages, and suggestions of online learning

**Fig. 1:**
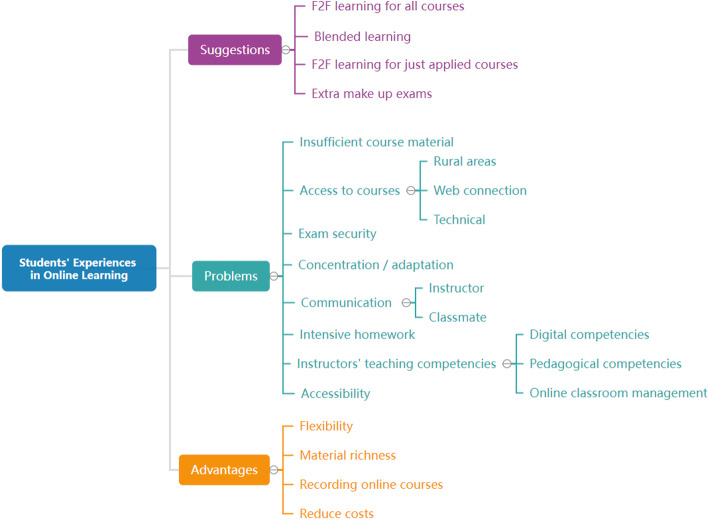
Suggestions, problems, and advantages of distance education

Students’ views on the distance education process examined and created advantages, problems, and suggestions of online learning categories. Figure 1 shows the findings for these three categories.

Based on Fig. 1, students’ suggestions for distance education mainly went back to face-to-face teaching. Moreover, these suggestions featured the inclusion of the blended learning model in the new education system, especially face-to-face (F2F) learning for just applied courses. Students who cannot attend the online exams also had requests about the extra make-up exam. Some of the suggestions offered by students in line with their online learning experiences are as follows;

*“I do not think that the courses I took during the distance education process were at all productive. Of course, while it would be wrong of me to say that I didn’t learn anything at all, it doesn’t offer the interaction that face-to-face education does.“ (Department of Computer and Instructional Technology Education, Junior)*

*“I want to go back to formal education as soon and as safely as possible. I can’t otherwise improve my skills required by my profession. I feel that my friends would agree with me.” (Department of Guidance and Psychological Counseling, Freshman)*

*“The pandemic rendered distance education mandatory. Model-wise, it has some highly successful advantages in both motivation and learning. That said, they should offer some courses both face-to-face as well as online.” (German Teaching, Freshman)*

*“It has as many pros as it does cons. If they were to implement a hybrid system combining the best of both distance and face-to-face education, it could lead to outstanding results.” (English Teaching, Sophomore)*

*“I’m majoring in the health department. Therefore, I don’t find distance education to be sufficient enough – especially since I wasn’t able to do any of the practices.” (Paramedic, Freshman)*

It was observed that the student’s views on the problems they experienced in the online learning process mostly focused on the inadequacy of the variety of materials offered in distance education. Also, they frequently mentioned the difficulties they encountered in accessing the courses, the lack of reliability in the exams, and the problems of focusing/adaptation in the courses. The problems such as the lack of communication between the students and the instructor and other students, the ineffectiveness of the applied courses, and the intensive homework of the instructors in the process were mostly reported. The problems the students expressed the least included the low digital competencies of the instructors and the failure to enter the exam grades on time. Some of the problems experienced by the students in line with their online learning experiences are as follows;

*“More synchronous and asynchronous resources should be provided. Our instructors should make the videos—as well as their asynchronous resources–.” (Department of Computer and Instructional Technologies Education, Senior)*

*“They should send us videos of the applied classes that we otherwise didn’t have the chance to take part in.” (Landscape Architecture, Freshman)*

*“Instructors who work with disabled students should be more careful and inclusive when teaching their courses. They should offer them a variety of materials that cater to their needs—that, to me, is very important.” (History, Freshman)*

*“Trying to reach/contact instructors—whatever the matter may be—is challenging. Even if they see your messages on OBS, they don’t reply. I’ve had so many problems with this.” (Child Development, Junior)*

*“Distance education isn’t sufficient enough when it comes to my learning. I can’t focus—especially when I’m at home, I can’t listen to anything with my family members around.” (Information and Document Management, Freshman)*

*“I had a lot of problems with distance education. I live in a rural area—I wasn’t able to take part in most of my courses/classes due to (lack of) internet access.” (Information and Document Management, Freshman)*

*“Only the copy groups successfully made it through distance education. Beyond that, students who couldn’t copy anything failed due to exams, which were (too) result-focused. All teachers were concerned about was preventing cheating. Their goal should have been to create exams that were fairer.” (Electrical-Electronics Engineering, Junior)*

The students’ views about the advantages offered by online learning were mostly observed on recording and re-watching the courses. Moreover, they frequently stated that online learning provided time/space flexibility and material diversity. The least expressed advantage by the students was the decrease in expenditures and expenses. Some of the students’ views on the advantages offered by online learning are as follows;

*“As someone who both works and studies at the same time, this opportunity greatly allowed me to use my time better.” (Astronomy and Space Sciences, Freshman)*

*“Distance education’s biggest pro was that it allowed me to watch my classes/courses when I wanted and for as long as I wanted. I was also able to choose at what time I did so according to when I was alert.” (Dentistry, Class 5)*

*“I realised that I could continue my education without having to be bound to (physical) spaces only. I feel that I’ll be able to make good use of that in my teaching career. It gave our teachers the chance to enrich their classes with diverse materials—which, in turn, allowed us to recognise our learning path out and create a suitable working plan around that.” (German Teaching, Junior)*

*“The videos our instructors made for (some of) our applied classes (which otherwise they would have had taught live/in-person) were great. They allowed me to understand processes in a more detailed fashion, and I could re-watch them.” (Computer Engineering, Junior)*

## Discussion and conclusion

The data for this study were collected at the end of the year when the COVID-19 pandemic emerged. When the data was collected, the courses in all universities were compulsory in distance education, asynchronous materials in the learning management system and synchronous live lessons. Within the scope of the present study, flexibility, self-regulated effort, and satisfaction in the online learning process of university students and their views on the distance education process were investigated.

The study found that the students’ flexibility of time management and flexibility of content levels were quite high. The students could structure their learning processes whenever, wherever, and for as long as they wanted in the online learning process. In terms of content flexibility, the students became a part of the learning process with their views, could rank the subjects according to their importance, choose where they would study, and learn the subjects grasping their interest. These results can be interpreted as the fact that the students adopted and applied the advantages of online learning, such as flexibility, time and place independence, repeatability of lessons and resources (Chow & Shi, 2014; Er Turkuresin, 2020; Turan & Gurol, 2020), which are stated in the literature. The students also mentioned the advantages of online education, such as flexibility, material richness, reduced costs, and recording of online courses. On the other hand, it was determined that the level of flexibility of teacher contact was relatively low. Therefore, it can be asserted that there are barriers to communicating with the instructors through different channels and whenever they want. This finding may be because teachers could not adequately respond to students’ questions or messages, except for synchronous live lessons. After all, teachers taught with an unfamiliar method during the pandemic. Likewise, previous studies reported that students had problems communicating with instructors (Sercemeli & Kurnaz, 2020; Tang et al., 2020). On the other hand, some results were also obtained, indicating that the instructors successfully managed the process, and the students could receive feedback when needed (Salturk & Gungor, 2020).

It was determined that the self-regulated efforts of the students in the online learning process were moderate. This result suggested that the students sometimes discontinued the work they planned to do without completing it due to losing focus; they gave up when the course was not understood and studied only on its easily understandable parts. In addition, they did not complete the subject when the instructional materials were complicated. However, based on this result, it can be interpreted that the online learning process that students experienced for the first time contributed to their self-regulation, albeit relatively. Students should have self-regulated learning skills to attend effectively learning activities in a flexible learning environment (Bergamin et al., 2012). Moreover, autonomous and flexible learning promotes self-regulation in students (Schraw, 2007).

This study found that students’ satisfaction with the distance education process was at a low level. However, in the literature, it has been stated that when various factors such as course resources, interaction, instructor satisfaction and evaluation are effectively structured in advance in distance education, students can mostly be satisfied with distance education (Baturay & Yükseltürk, 2015; Harsasi & Sutawijaya, 2018; Şahin, 2009; Secreto & Pamulaklakin, 2015; Moore, 2002). The reason for this finding may be that many face-to-face courses are urgently converted to distance education with the COVID-19 pandemic, and students are negatively affected by these complex and stressful processes (Atasoy et al., 2020; Lee et al., 2021; Sercemeli & Kurnaz, 2020; Şimşek et al., 2021; Tang et al., 2020; Turan & Gürol, 2020). Students experience difficulties such as independent learning, time management, and maintaining motivation in the online learning process and problems such as accessibility, digital division, and inequality (Lee et al., 2021; Shin & Hickey, 2020). The qualitative findings indicated limitations such as the problem of accessing the courses due to the technical infrastructure, the increased workload, the lack of communication with the instructor and peers, and insufficient instructional materials.

It was determined that male students’ flexibility, self-regulated effort, and satisfaction levels were higher than their female counterparts. There was a significant difference in flexibility of teacher contact and satisfaction, but the effect values were low. The literature includes results reporting that satisfaction does not change according to gender, and even though there is a significant difference, the effect size is small (Er Turkuresin, 2020; Simsek et al., 2021). Upon the examination made in terms of departments/disciplines in the study, it was seen that the flexibility, self-regulated effort, and satisfaction levels of the students studying in the discipline of education were significantly higher than the other disciplines in general, although the effect size was small. This finding can be explained by the fact that faculty members of the faculty of education adapted to the online education process more quickly and managed it well due to their pedagogical knowledge. On the other hand, parallel with Simsek et. al.’s (2021) study results, engineering and natural sciences students were relatively more satisfied with distance education. Also, similar to previous studies, students studying in the applied sciences, such as medical and health sciences departments, had relatively low satisfaction with online learning (Aristovnik et al., 2020; Atilgan et al., 2021).

There were moderate correlations between flexibility, self-regulated effort, and satisfaction. This result can be interpreted as the fact that these variables were crucial in structuring the effective online learning process. Satisfaction is one of the critical factors in determining the effectiveness of the online education process and is intertwined with many other factors (Hamdan et al., 2021; Wei & Chou, 2020). In this study, it was revealed that flexibility and self-regulated effort significantly predicted satisfaction. The variable of self-regulated effort had the most important effect on satisfaction, and this variable was followed by the flexibility of the time management variable. Distance education requires students to act more autonomously and take more responsibility for regulating learning processes to achieve their learning goals (Shearer & Park, 2018). Students with high self-regulation skills are active participants in their metacognitive, motivational, and behavioural processes (Zimmerman, 1990). Studies in the literature have reported that self-regulation is an important variable that predicts student satisfaction and success (Barak et al., 2016; Cho & Kim, 2013; Hamdan et al., 2021; Inan et al., 2017; Ng & Baharom, 2018; Yavuzalp & Bahcivan, 2021). On the other hand, some other studies have stated that students’ finding online courses flexible in distance education has important effects on their satisfaction levels (Chow & Shi, 2014; Sahin & Shelley, 2008). It can be asserted that flexibility provides satisfaction with online learning by addressing the needs of working and adult students (Ammenwerth et al., 2019) since it considers the diversity of students’ knowledge and life experiences and supports self-learning.

## Limitations, implications, and future research

In this study, the university students’ flexibility, self-regulated effort, and satisfaction levels in the distance education process were examined in detail, and important results were revealed. The data were collected from a high number of students in the study, which increased the generalizability of the results. However, limitations of the study are that the study was conducted in the context of universities in Turkey and TRNC, using a convenience sampling method, by collecting data with self-reported data. Another limitation of this study is the possibility that the crisis environment created by the pandemic may affect the findings since the data were collected during the distance education process carried out during the COVID-19 pandemic period. Based on the study results, it can be said that universities should develop strategies based on disciplines to increase the satisfaction of students when they have to take the necessary courses due to the pandemic process and offer education entirely online. Additional support services can be provided to the instructors so that the student–teacher interaction is not interrupted during the distance education process. In-service training can be organised to improve the distance education competencies of instructors. For increasing students’ self-regulation skills, students can be supported in this process by organising specific activities in classes starting from primary school. A hybrid approach can be adopted, especially in applied courses. Universities should go into a restructuring process according to their conjunctures and establish sustainable digital transformation strategies to increase the effectiveness of online education. Path analysis studies can be carried out by examining other variables affecting distance education satisfaction in future studies. In addition, the students’ self-regulated learning skills can be modelled by examining the log data on their teaching systems.

## Data Availability

The datasets used and/or analysed during the current study are available from the corresponding author on reasonable request.

## Abbreviations

COVID-19: Coronavirus disease 2019
TRNC: Turkish Republic of Northern Cyprus
FTM: Flexibility of time management
FTC: Flexibility of teacher contact
FC: Flexibility of content
SRE: Self-regulated effort
SAT: Satisfaction
F2F: Face-to-face
